# Natural and Synthetic Saponins as Vaccine Adjuvants

**DOI:** 10.3390/vaccines9030222

**Published:** 2021-03-05

**Authors:** Pengfei Wang

**Affiliations:** Department of Chemistry, University of Alabama at Birmingham, Birmingham, AL 35294, USA; wangp@uab.edu

**Keywords:** adjuvant, saponin, mechanism, SAR, QS-21, VSA-1, VSA-2

## Abstract

Saponin adjuvants have been extensively studied for their use in veterinary and human vaccines. Among them, QS-21 stands out owing to its unique profile of immunostimulating activity, inducing a balanced Th1/Th2 immunity, which is valuable to a broad scope of applications in combating various microbial pathogens, cancers, and other diseases. It has recently been approved for use in human vaccines as a key component of combination adjuvants, e.g., AS01b in Shingrix^®^ for herpes zoster. Despite its usefulness in research and clinic, the cellular and molecular mechanisms of QS-21 and other saponin adjuvants are poorly understood. Extensive efforts have been devoted to studies for understanding the mechanisms of QS-21 in different formulations and in different combinations with other adjuvants, and to medicinal chemistry studies for gaining mechanistic insights and development of practical alternatives to QS-21 that can circumvent its inherent drawbacks. In this review, we briefly summarize the current understandings of the mechanism underlying QS-21’s adjuvanticity and the encouraging results from recent structure-activity-relationship (SAR) studies.

## 1. Introduction

Traditional vaccines are whole-organism-based, using live attenuated or inactivated viruses or bacteria. These vaccines are quite reactogenic due to the presence of numerous pathogen-associated-molecular-patterns (PAMPs) that are responsible for activation, and sometimes over-activation, of the immune system. While being effective, the traditional vaccine development approach is inapplicable to combat against not only many other severe infection diseases, but also cancers and chronic inflammatory and autoimmune diseases [1,2,3,4,5]. The emergence of new pathogens and inadequate protection conferred by some of the existing vaccines, especially in certain age groups or in immunocompromised individuals highlights the need for more sophisticated and effective vaccines. One of the important developments in the field is subunit vaccines that employ well-characterized, highly purified, and safer antigens such as recombinant proteins and peptides. Subunit vaccines not only reduce side effects, but also provide a platform for exploring molecular mechanisms of the immune response. However, their highly refined antigens (thus with limited or no PAMPs) do not activate as many facets of the immune response as whole-organism-based vaccines. The poor immunogenicity generally leads to stimulation of a weak and short-lived immunity. To boost immunogenicity, it is crucial for subunit vaccines to have immunostimulatory adjuvants that enhance immune responses induced by the poorly immunogenic antigens.

Vaccine adjuvants are non-immunogenic substances that can improve or modulate antigen-specific immune responses toward their co-administered antigens, constituting an indispensable element of modern vaccines [6,7,8,9,10,11,12,13,14,15,16,17,18,19]. They can (a) enhance the ability of a vaccine to elicit strong and durable immune responses (including in immunologically compromised individuals such as immunologically immature (neonates), aged, and immune suppressed individuals) [20], (b) reduce antigen dose and the number of immunizations, and (c) modulate the nature of immune response (e.g., in favor of humoral or cell-mediated response). Despite the increasingly important role of adjuvants, there only a few adjuvants approved for human use [15,18,19,21,22]. Alum (various aluminum salts), the first and most commonly used adjuvant, had been the only human vaccine adjuvant for more than nine decades until 2009 [23,24,25]. It primarily enhances Th2 humoral responses, effective for neutralizing vaccines, but ineffective for vaccines targeting intracellular pathogens or cancer cells when a Th1 or mixed Th1/Th2 response is required. Moreover, alum is not compatible for mucosal vaccines. In 2009, Food and Drug Administration (FDA) approved AS04, a combination of alum and monophosphoryl lipid A (MPLA), for human use [26,27]. MPLA was the first Toll-like receptor (TLR) agonist approved for human vaccine. Since 2016, FDA has approved three more adjuvants (i.e., MF59/AS03, CpG 1018, and AS01b). MF59/AS03 is a squalene-based oil-in-water emulsion used in influenza vaccines [28,29,30]. CpG 1018 is a TLR9 agonist used in a Hepatitis B vaccine; it is a short, 22-base synthetic oligonucleotide mimicking bacterial and viral genetic material [31,32]. AS01b was approved recently for use in Shingrix^®^ against herpes zoster by FDA [33,34] and Mosquirix^®^ against malaria by European Medicines Agency (EMA) [35]. It is a combination adjuvant containing MPLA and saponin QS-21 in a liposomal formulation that induces strong humoral and cellular immune responses [33,36].

## 2. Immunostimulatory Adjuvant QS-21

**Figure 1:**
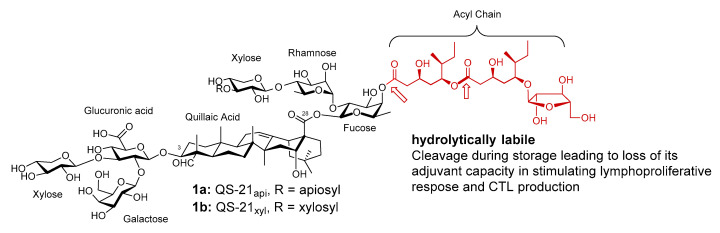
Natural saponin adjuvant QS-21.

QS-21 is a mixture of two isomeric bidesmosidic saponins (1, Figure 1), isolated from the tree bark of *Quillaja saponaria* Molina (QS), an evergreen tree native to temperate central Chile. It can potentiate a balanced Th1/Th2 response with antigen-specific cytotoxic T lymphocyte (CTL) production [37,38,39,40].

QS-21 has a triterpene aglycone core, quillaic acid, with a branched trisaccharide connecting to the C3 OH group through a beta glycosidic ether bond, and a linear tetrasaccharide connecting to the C28 carboxyl group through a beta glycosidic ester bond (Figure 1). The reducing end of the C28 tetrasaccharide is a *β*-d-fucosyl unit with its 4-O position capped with a glycosylated pseudodimeric fatty acyl chain. The two structural isomers of QS-21 only differ in the nonreducing end of the C28 tetrasaccharide, i.e., QS-21_api_ (~65% abundance) has a terminal *β*-d-apiosyl unit while QS-21_xyl_ (~35% abundance) has a terminal *β*-d-xylosyl unit. The structure of the two isomers were confirmed by Gin et al. with organic synthesis [41,42]. The two isomers have similar adjuvant activity and toxicity [43].

QS-21 is amphipathic with its hydrophobic (triterpene core and fatty acyl chain) and hydrophilic (sugar) moieties. The amphipathicity can cause permeation of cell membranes, which was attributed to its intrinsic lytic activity and cytotoxicity [39,44,45,46]. Melzig et al. suggested that the general cytotoxicity of saponins was mainly dependent on their membrane toxicity, which might be caused by the loss of cholesterol from the cell membrane [47]. The lytic activity of QS saponins can be reduced significantly through proper formulation, such as in liposomes in the presence of cholesterol (e.g., AS01), in emulsions (e.g., AS02), or in ISCOMATRIX (IMX) with phospholipid and cholesterol [48,49,50]. It was suggested that saponins might interact with membranes in a cholesterol-dependent manner, leading to pore formation [51,52]. Korchowiec et al. recently demonstrated that two structurally different saponins, digitonin and Merck saponin, showed high affinity for cholesterol in lipid model membranes by using Langmuir monolayer techniques combined with polarization modulation infrared reflection-absorption spectroscopy (PM-IRRAS) and Brewster angle microscopy [53]. However, amphipathicity is probably not responsible for QS-21’s unique adjuvant activity in that most of amphipathic saponins do not have QS-21’s superb immune stimulating activity [54]. QS-21’s amphipathicity can also lead to micelle formation in aqueous solutions [55], for example, it has a critical micelle concentration (CMC) at 51 µg/mL in succinate buffered aqueous solutions. However, QS-21’s adjuvanticity seems to be irrelevant to micelle formation in that it remains active at concentrations below the CMC [56].

**Figure 2:**
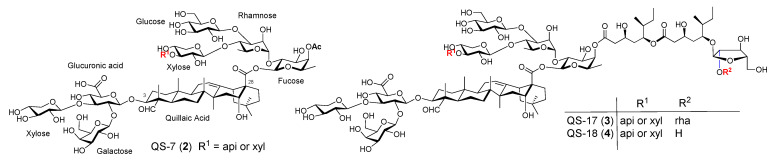
Natural *QS* saponins QS-7, QS-17, and QS-18.

Along with QS-21, there are three other structurally characterized saponins in the same tree bark extract, i.e., QS-7 (**2**), QS-17 (**3**), and QS-18 (**4**) (Figure 2) [37,39]. QS-7 (**2**, Figure 2) has adjuvant activity similar to QS-21 but lower toxicity [57]. Its structure is significantly different from the other three characterized QS saponins: It does not bear a glycosylated pseudodimeric acyl side chain in the C28 oligosaccharide domain. Gin et al. confirmed the structure of natural QS-7 via organic synthesis in 2008 [58]. The respective fatty acyl chain of QS-17, 18, and 21 was believed to be responsible for their capability of stimulating cellular immunity [55,59]. QS-18 is the most abundant saponin in the QS Molina tree bark extract. The main structural difference between QS-17/18 and QS-21 is in the C28 oligosaccharide domain. Instead of having a linear tetrasaccharide as in QS-21, QS-17/18 has an additional *β*-d-glucopyranosyl (glc) unit connected to the *α*-L-rhamnopyranosyl (rha) unit at its 3-O position. QS-17 differs from QS-18 only in the R^2^ group of the acyl side chain, i.e., QS-17 has a disaccharide unit while QS-18 has a monosaccharide unit at the far end of the side chain (Figure 2). These three QS saponins have a similar adjuvant activity profile but QS-17 and QS-18 are more toxic than QS-21. The higher toxicity of QS-18 than that of QS-21 was attributed to the extra glc branch in the C28 oligosaccharide domain; however, QS-7, with the same branch at the same site, is notably less toxic than QS-21. These observations suggest that immunostimulating activity and toxicity of these saponins are probably determined by each individual saponin’s specific molecular structure instead of by the presence/absence of a certain structural feature in the saponin or by its amphipathicity.

QS-21 has been extensively studied in the form of a combination adjuvant [60,61,62,63,64,65]. It is a key component of the GlaxoSmithKline’s Adjuvant System AS01 (including the FDA-approved AS01b), AS02 (an oil-in-water emulsion containing QS21 and MPL), AS05 (liposomes containing QS21, MPL, and alum), and AS15 (liposome formulation containing QS-21, MPL, and CpG) [34,63,64,65,66,67,68,69,70]. These systems are effective in enhancing both humoral and cellular immunogenicity of different types of antigens in human subjects [66,69] and can also be effective for certain patient populations [67]. The QS-21-containing combination adjuvants have been recently reviewed [26,33,36], and they will not be discussed in this review.

## 3. Mechanism Studies of QS-21

In general, vaccine adjuvants can first help to create a proper local immune environment at the injection site(s) by activating innate immune responses, which are responsible for the development of the subsequent adaptive immune responses [12,71]. Currently known mechanisms of action of adjuvants include (a) sustaining release of antigens at the injection site, (b) modulating production of cytokines and chemokines, (c) recruiting of immune cells to the injection site to create a proper local immune environment, (d) upregulating the expression of Major Histocompatibility Complex (MHC)-I and/or MHC-II and co-stimulatory signals CD40, CD80/86 on the surface of antigen-presenting cells (APCs), (e) enhancing antigen processing and expression by APCs, (f) facilitating migration of the mature APCs to the draining lymph nodes (dLNs), (g) helping activation of antigen-specific B or T cells through their interaction with mature APCs to produce proper B cells, CD4+, and/or effector CD8+ T cell responses [72].

The mechanism underneath QS-21′s adjuvanticity remains largely unknown despite its wide use in various experimental vaccines and its approval for clinic use [35]. It is unclear whether QS-21 activates immune cells via a specific receptor and what particular signaling pathways are responsible for its extraordinary immunostimulating activity. However, efforts in elucidating its mechanism have led to valuable insights.

It is known that QS-21 does not operate by a depot effect to extend presentation of the co-administered antigen to the immune system [37]. Reimer et al. used an adjuvant formulation containing natural QS saponins, i.e., Matrix-M^TM^, to study its role in stimulating immune responses in BALB/c mice [73]. Matrix-M^TM^ is comprised of 40 nm nanoparticles made of *Quillaja* saponins, cholesterol, and phospholipids [50,74,75]. In the absence of antigen, the adjuvant led to a local transient immune response with recruitment and activation of central immune cells to dLNs 48 h post subcutaneous injection. These effects might play a role in enhancing uptake and presentation of vaccine antigens to elicit a competent immune response. By using synthetic QS-21 analogs as molecular probes, Tan, Lewis, and Ragupathi et al. suggested their synthetic QS-21 analogs had a potential role in facilitating antigen trafficking by APCs from the site of injection to the dLNs, where the processed antigens are presented to the adaptive immune system [76]. However, the molecular structure of the employed saponin probes is significantly different from that of QS-21, and it is unclear whether the synthetic analogs inherited the same adjuvant activity profile and thus the same mechanism of QS-21. Thus, the observations with the synthetic probes might not be extrapolated to natural QS-21.

Newman et al. showed through in vivo and in vitro cell depletion and reconstitution studies that macrophages could be an important site of action for QS-21 [77]. Recently, by using QS-21 formulated in liposomes with Hepatitis B surface antigen (HBsAg) or ovalbumin (OVA) antigen, Goriely and Didierlaurent et al. observed that intramuscular immunization in mice led to a rapid local innate response in the dLN. They identified CD169+ resident macrophages of the lymph node draining the injection site as the main cells targeted by QS-21 in formulation with liposomes. QS-21 accumulated rapidly in CD169+ resident macrophages of the dLN, where it induced Caspase-1 activation and caused the release of high-mobility group protein B1 (HMGB1). These early events led to the recruitment of innate immune cells and activation of dendritic cells (DCs) which then presumably triggered MyD88-dependent activation of antigen-specific cellular and humoral responses. The adjuvant effect of QS-21 depended on the integration of Caspase-1 and MyD88 pathways, at least in part through the local release of HMGB1 [78]. Depletion of these CD169+ resident macrophages abrogated QS-21-mediated innate cell recruitment to the lymph node, DC phenotypic maturation as well as the adjuvant effect on T-cell and antibody responses to co-administered antigens.

Regarding DC activation, it is known that QS-21 does not bind to Toll-like receptors 2 and 4 [79]. Marciani et al. suggested that QS-21 would be unlikely to activate DCs by interacting directly with specific cell receptors. Instead, *Quillaja* saponins (including QS-21) probably could act on DCs in a non-receptor-mediated manner [80]. QS-21 could facilitate the entry of exogenous protein antigens into DCs by cholesterol-dependent endocytosis [80]. It then would disrupt the endosomal membrane to facilitate early escape of the antigens for further processing inside the cell. The processed antigen fragments would be loaded into MHC I and cross-presented on the DC surface to initiate production of CTLs.

Goriely et al. recently reported that QS-21 directly activated human monocyte-derived dendritic cells (moDCs) and promoted a pro-inflammatory transcriptional program [81]. Thus, QS-21 entered DCs via a cholesterol-dependent endocytosis, and then destabilized lysosomes through pore formation, causing release of their contents including lysosomal enzymes, which resulted in inflammasome activation, Syk- (a tyrosine kinase) and cathepsin-B- (a cysteine protease) dependent cell activation and cytokine production in moDCs. Their results suggested that cytokine production by QS-21-stimulated moDCs did not depend on inflammasome activation. However, some results were drawn using molecular probes such as BODIPY-QS-21 (a boron-dipyrromethene and QS-21 conjugate) and ^14^C-QS-21 (structure unrevealed) whose adjuvant activities were not fully characterized against QS-21. It is known that structural modification of QS-21 could significantly alternate its adjuvant property and thus mechanism. In order to ensure their results relevant to QS-21, it is important to confirm that the adjuvant activity profiles of the synthetic probes closely resemble that of QS-21. In a related study, Melzig et al. analyzed the influence of a significantly membrane toxic saponin on the cholesterol content of intracellular membranes such as those of endosomes and lysosomes, and found that a membrane toxicity mechanism featuring the saponin-dependent loss of membrane cholesterol was not detectable on the endosome/lysosome membrane [47].

Adema et al. noticed saponin adjuvants could induce cross-presentation in DCs by intracellular lipid body (LB) formation [82], which could be relevant to QS-21’s mechanism. LB organelles consist of a phospholipid monolayer that contains numerous proteins and a core of neutral lipids, such as sterol esters or triacylglycerols. *QS* saponins (containing QS-21) in the form of ISCOM induced intracellular LB formation only in the CD11b+ DC subset in vitro and in vivo. LB formation was positively related to the enhanced cross-presentation of antigens and T-cell activation [83]. LBs facilitated the proteasomal route of cross-presentation and blockade of LB induction effectively abrogated the saponin-induced antigen cross-presentation.

Inflammasomes are an essential part of the innate immune system and their activation can stimulate the adaptive immune system; thus, they are often the targets for vaccine adjuvants [84]. The role of QS-21 in activating NOD-like receptor protein 3 (NLRP3) inflammasome seems to be complicated. On one hand, Lien et al. observed that QS-21 itself did not activate NLRP3 inflammasome and the subsequent production of cytokines IL-1β or IL-18 in bone marrow-derived dendritic cells (BMDCs) or immortalized mouse macrophages, unless paired with MPLA. Moreover, NLRP3-deficient mice immunized with HIV-1 gp120 and QS-21 showed significantly higher levels of Th1 and Th2 antigen-specific T cell responses and higher IgG1 and IgG2c activities than the wild-type controls [85]. On the other hand, Goriely and Didierlaurent et al. observed QS-21 formulated in liposomes induced NLRP3 inflammasome activation and Caspase-1-dependent production of IL-1β, suggesting that NLRP3 activation was involved in QS-21-induced immune responses such as innate cell recruitment and both CD4 and CD8 T-cell responses [78].

In a related study, Morelli and Schnurr et al. evaluated immune cell responses to *Quil* A (purified *QS* saponins, containing QS-21) in the form of IMX in mice [86]. IMX is comprised of phospholipid, *Quil* A, and cholesterol components that form cage-like structures ∼40 nm in diameter [48]. The complex was identified as a potent activator of the NLRP3 inflammasome in APCs in vitro, leading to IL-1β and IL-18 production; however, both inflammasome-related and -unrelated pathways contributed to IL-18-dependent IMX-mediated NK cell activation and vaccine-induced cytotoxic T cells and B cell immunity in vivo. NLRP3 was dispensable for induction of NK or T cell immune responses.

Regarding activation of T cells, Kensil et al. postulated that QS-21 might stimulate T cells via receptor-mediated mechanisms [87,88]. They suggested that QS-21 could build a chemical bond by using its carbonyl group of the quillaic acid triterpene core to react with an amino group of putative T-cell surface receptors. This chemical event could provide co-stimulation signals for T-cell activation, an analogy to the previous observation with tucaresol, which is a carbonyl-containing small molecule, believed to send costimulatory signals to the CD4+ T cell via the chemical event of forming Schiff base on T-cell surface amines through its aldehyde moiety [89]. In the presence of tucaresol, stimulation of the T cells with anti-CD3 led to a five- to tenfold enhancement of IL-2 production. However, there lacks direct experimental evidence to confirm that QS-21 can effectively form Schiff base on T cell surface, albeit there is indirect evidence from the studies of Kensil et al. showing that chemical modification of the quillaic acid carbonyl group resulted in severely diminished adjuvant activity of QS-21 [88].

## 4. Structure-Activity-Relationship (SAR) Studies of QS-21

QS-21 has been proved to be a unique and potent adjuvant for applications in clinical and experimental vaccines, even though its detailed cellular and molecular mechanisms remain largely unknown. Parallel to the efforts of expanding its application and exploring its mechanism, extensive medicinal chemistry efforts have been devoted to its SAR studies, aiming at gaining mechanistic insights, and developing molecular probes and practical QS-21 alternatives that are devoid of its inherent drawbacks [19,90]. For example, abundance of QS-21 in *QS* tree bark extracts is low [91], and overexploitation of the natural source has resulted in ecological damage and shortage of available supplies even under the current demand, leading to more strict environmental regulations and increased price [92]. It was estimated that the current global supply of natural QS-21 was only enough for approximately 6 million doses (100 μg/dose for human use) [93], not sufficient for widespread clinical use for various anti-infective vaccines. QS-21′s adjuvanticity increases with dose, however, its dose-limited side effects prevent it from reaching its full potential. Early SAR studies indicated that adjuvanticity and toxicity of *QS* saponins could be decoupled, and toxicity of *QS* saponins could be independently modulated through specific structural modifications while maintaining potent adjuvant activity [94,95]. The limited supply of QS-21, along with its dose-limiting toxicity, chemical instability, and laborious and low-yielding purification, hinder its wider use [92,93]. There is an imperative need for potent and practical alternatives to QS-21 that are more potent and less toxic [96].

It was suggested that QS-21 could interact with immune cells in a receptor-mediated or a non-receptor-mediated manner. SAR studies indicate a receptor-mediated interaction could play a crucial role in that saponins with the same or similar hydrophile−lipophile balance (HLB), but only subtle structural differences show significantly different adjuvant activities.

**Figure 3:**
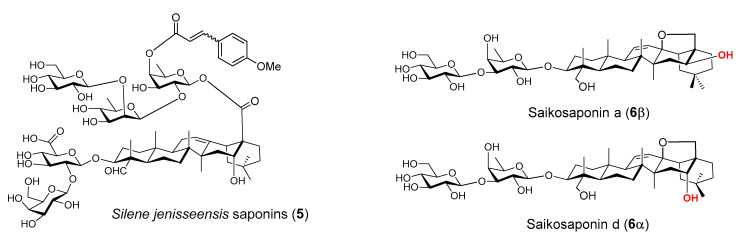
Immunostimulating natural saponins.

Some structural features of QS-21 have been identified as being relevant to its adjuvant activity, e.g., the C23 aldehyde moiety of the quillaic acid core and the fatty acyl side chain that caps O-4 of the fucosyl unit at the reducing end of the C28 oligosaccharide. It has been postulated that the carbonyl group forms a Schiff base with an amino group of putative T-cell surface receptors to provide co-stimulation for T-cell activation [80,89]. The hypothesis is supported by the observations that (a) saponins lacking such an aldehyde group are devoid of these activities, and (b) chemical modification of the aldehyde group results in loss of adjuvant activities. Moreover, some other saponins bearing a carbonyl group in their respective structure also show immunostimulating properties, e.g., gypenosides from the Cucurbitaceae *Gynostemma pentaphyllum*, and curculigosaponin G from the rhizomes of *Curculigo orchioides* [97]. However, there are also plenty of saponins capable of stimulating immune responses, including cellular responses in the absence of such a carbonyl group, e.g., ginsenosides [97]. There are also saponins that have structure similar to that of QS-21, but show no immunostimulating properties. For example, the triterpene glycosides (**5**, Figure 3) isolated from *Silene jenisseensis* (Caryophyllacea) [98] have the identical quillaic acid triterpene core to QS-21, and similar to QS-21, it has a fatty acyl group (i.e., *trans- or cis*-*p*-methoxycinnamoyl group) on the fucose residue of the C28 oligosaccharide reducing end. These examples suggest that it is probably the specific structure of the whole saponin molecule that determines its adjuvant properties. Structurally similar saponins with the “key” functional groups of QS-21, e.g., C3 oligosaccharide, C16 hydroxyl group, C23 carbonyl group, C28 oligosaccharide, and its reducing end fucosyl unit capped with a fatty acyl chain at 4-O, will not be warranted with adjuvant activities similar to that of QS-21. Another relevant evidence supporting that saponins’ adjuvant activity may rely on receptor-mediated interactions rather than saponins’ amphiphilicity or their possession of certain functional groups was demonstrated by saikosaponins (**6**, Figure 3). Saikosaponins have a stimulatory effect on macrophage activation, with saikosaponin-d (**6α**) being more effective than saikosaponin-a (**6β**). These two saponins differ only in their stereo-configuration at C-16 [99].

**Figure 4:**
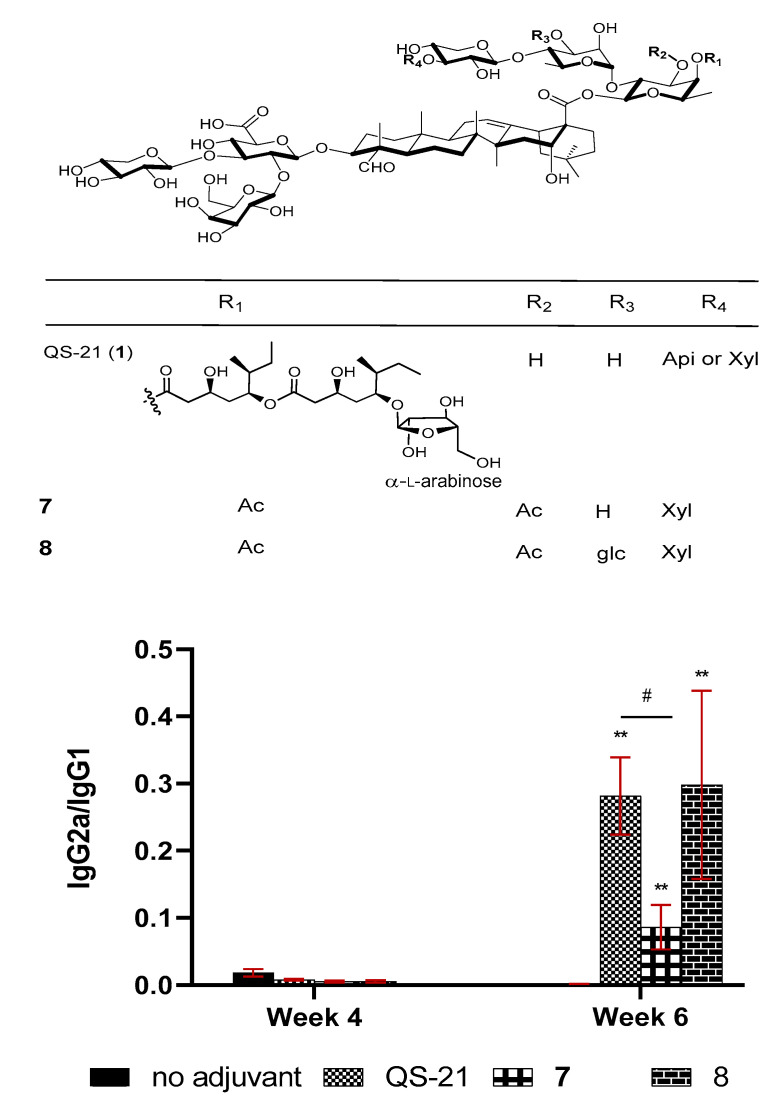
Groups of female BALB/c mice were immunized by the subcutaneous route (s.c.) with OVA (20 µg) alone or with QS-21 (20 µg), or a synthetic saponin (50 μg). IgG2a/IgG1 ratios are expressed as mean ± SEM. Statistical significance was evaluated by *t* tests (with unpaired, nonparametric, and Mann-Whitney test). ** *p* < 0.01 compared with the group of OVA alone, and # *p* < 0.05 compared between the indicated groups.

QS-21 is one of the rare saponins bearing an acyl side chain. The specific role of the acyl group remains unclear, but SAR studies proved that it was crucial to the potent activity of QS-21 in stimulating a Th1 immunity [55,59]. Removal of the acyl group led to QS-21’s loss of its ability in stimulating a lymphoproliferative response and CTL production, but the deacylated QS-21 remained active in potentiating Th2 responses [59,100,101,102,103]. It was postulated that by removing the acyl group, the reducing end fucosyl unit of the C28 oligosaccharide would be exposed for its putative interaction with dendritic cell-specific intercellular adhesion molecule-3-grabbing non-integrin (DC-SIGN) receptors on DC, inducing a Th2-skewed immunity [104]. It remains to be proven that the internal fucosyl unit is capable of binding to DC-SIGN, which is only known for binding with a terminal fucosyl unit or other types of sugars [105,106,107,108,109,110,111]. Even if the presumed binding event is responsible for the Th2-only immunity induced by the deacylated QS-21, the fatty acyl chain of the natural QS-21 is more than just blocking its binding with DC-SIGN. In fact, synthetic QS analog **7** (Figure 4) with the natural acyl side chain replaced with a simple acetyl group should still block the fucosyl/DC-SIGN binding; however, it showed inferior adjuvant activity to that of QS-21 and a much lower IgG2a/IgG1 ratio (indicating its induction of a less Th-1 skewed immunity) [112]. Since production of IgG1 or IgG2a in mice is enhanced by the respective Th2 or Th1 cytokines, the relative amount of IgG2a and IgG1 was often used as a tentative indication of involvement of Th1 and Th2 immunity potentiated by the adjuvant. Interestingly, by adding one glc unit to saponin **7** at the particular position boosted adjuvant activity of the resulting new saponin **8** (structurally resembling QS-7 more than QS-21) in terms of enhancing IgG, IgG1, and IgG2a production to the same level as QS-21, and a similar IgG2a/IgG1 ratio as well (Figure 4) [112].

Marciani et al. developed the semi-synthetic saponin analog GPI-0100 [100,101,102,103,113]. It was prepared from *Quil* A, a purified mixture of complex QS tree bark extracts (containing QS-17, -18, -21, and numerous other saponins), by complete removal of the acyl side chain and subsequent incorporation of a dodecylamine chain via amide formation. The resulting complex mixture, GPI-0100, retains the capacity of stimulating humoral and cellular immunity. It is 20 times less lethal in mice than QS-21. In a study of a prostate cancer vaccine, GPI-0100 was found to be more potent than QS-21 in mice at doses that were less toxic [114]. The immunological property of GPI-0100, especially its retained capacity of stimulating a cellular immunity, indicated the important role of a side chain in inducing a Th1 immunity. It also indicated that it was not crucial to have the side chain cap the fucosyl unit as in natural QS-21.

**Figure 5:**
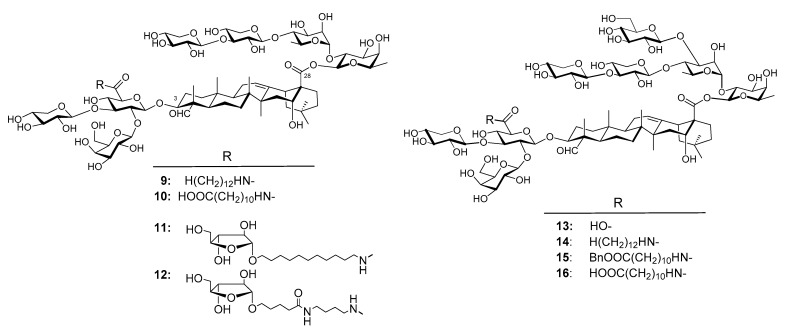
Synthesized *QS* analogs.

Wang et al. synthesized unnatural analogs of QS-21, e.g., **9**–**12** (Figure 5) [115,116]. Similar to GPI-0100, they have an aliphatic chain connected to the C3 trisaccharide domain through a chemically stable amide bond instead of an acyl side chain as in QS-21. The results showed that saponins **10**–**12**, and especially **10** and **11**, were effective in potentiating a serum antigen-specific IgG response following systemic immunization [80]. Analysis of the IgG2a/IgG1 ratio of the antigen-specific responses indicated that GPI-0100, **10**, and **11** potentiated mixed Th1 and Th2 antigen-specific responses, whereas **12** mainly enhanced Th2 responses. Taken together, the results demonstrated that QS-21 analogs **10** and **11** were comparable to GPI-0100 in potentiating systemic antigen-specific responses. The results confirmed that derivatization at the glucuronic acid unit attached to C3 of the triterpenoid core could be a viable way to access chemically stable new saponin adjuvants, as suggested by the early SAR studies of Kensil et al. [88] and the preclinical success of GPI-0100. It is a practical and cost-effective strategy for preparing a large number of saponin adjuvants for property screenings and large-scale production [115,116,117]. The results also clearly showed that the structure of the incorporated side chain had a significant impact on adjuvant activity in terms of the magnitude and nature of the responses [116].

Wang et al. also synthesized QS-17/18 analogs, e.g., **13**–**16** (Figure 5) [117,118]. Analogs **14** does not have the natural acyl side chain at the original position, instead it has a plain aliphatic side chain on the other side of the molecule. By using the same immunological evaluation procedure for QS-21 analogs **9**–**12**, the results showed that analog **13** without a side chain had a significantly different adjuvant activity profile from that of **14**, especially in boosting IgG2a responses. Saponin **13** only stimulated a Th2 immune response, while analog **14** had an adjuvant activity profile similar to that of GPI-0100 and thus might potentiate a mixed Th1 and Th2 immunity [118]. Saponin **15** stimulated a more Th2-skewed immunity, and **16** stimulated a more Th1-skewed immunity than other QS-17 analogs [117,119].

**Figure 6:**
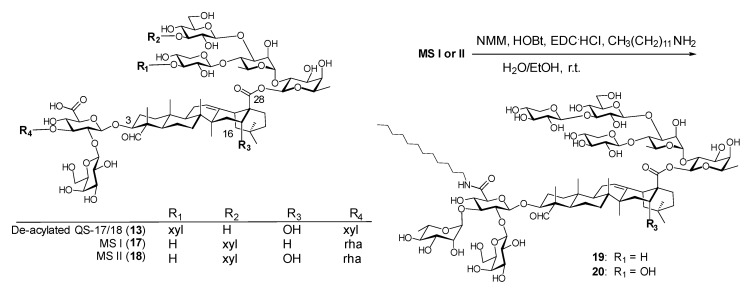
Structural similarity between *Momordica* saponins (MS) and de-acylated QS-17/18 and MS derivatization.

Wang et al. noticed the strikingly structural resemblance between the de-acylated QS-17/18 (**13**) and *Momordica* saponins (*MS*) I and II (**17** and **18**, Figure 6). These two saponins were isolated from the seeds of *Momordica cochinchinensis* SPRENG (MC), a perennial vine with readily available seeds [120]. Their structures only differ in the triterpenoid core, i.e., *MS* I (**17**) has a gypsogenin core (R_3_ = H, Figure 6) while *MS* II (**18**) has a quillaic acid core (R_3_ = OH, Figure 6), identical to the core of QS-21 and QS-17/18. Based on the different adjuvant properties observed in saponins **13** and **14**, it was inferred that incorporating the same plain side chain of **14** to *MS* I/II might lead to new adjuvants with similar adjuvant activity to **14** due to their structural resemblance. Thus, saponins **19** and **20** were readily synthesized in one step as depicted in Figure 6.

**Figure 7:**
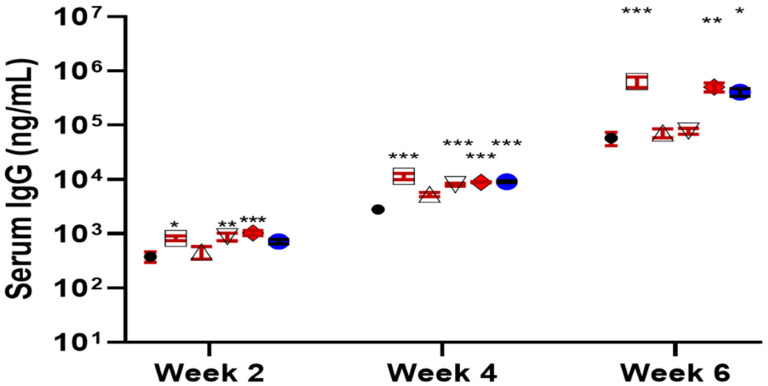

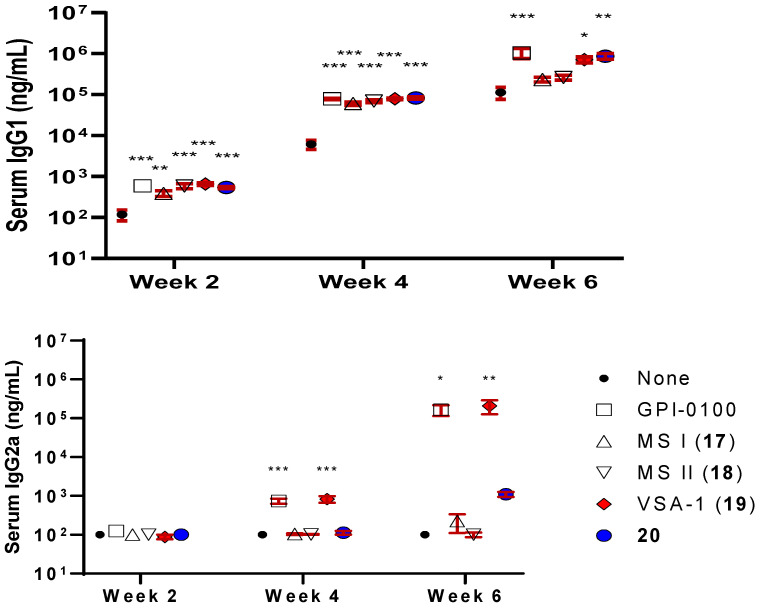
Serum IgG, IgG1, and IgG2a anti-OVA response in mice immunized by the s.c. route with OVA alone or with GPI-0100 or a saponin adjuvant. Mice were immunized on days 0, 14, and 28. Serum samples were collected prior to each immunization and at 6 weeks following the initial immunization. Values are expressed as mean ± SEM. * *p* < 0.05, ** *p* < 0.01, and *** *p* < 0.001 compared with mice immunized with OVA alone.

Indeed, the new derivatives, **19** and **20**, showed significantly improved adjuvant activity in terms of antigen-specific IgG secretion when compared with the natural *Momordica* saponins (Figure 7). It was expected that the derivative of *MS* II should resemble the adjuvant property of **14** more than that of *MS* I in that both *MS* II and *QS* derivatives have the same quillaic acid triterpene core while *MS* I has a gypsogenin core. Interestingly, it was the derivative of *MS* I, i.e., **19** (named VSA-1), showed an IgG2a/IgG1 ratio similar to that of **14** and the positive control GPI-0100 (Figure 7). Wang et al. went on to confirm that in the *MS* series of derivatives, the structure of the incorporated side chain has a significant impact on adjuvanticity [119], as seen in the QS series of derivatives [95,116,119].

**Figure 8:**
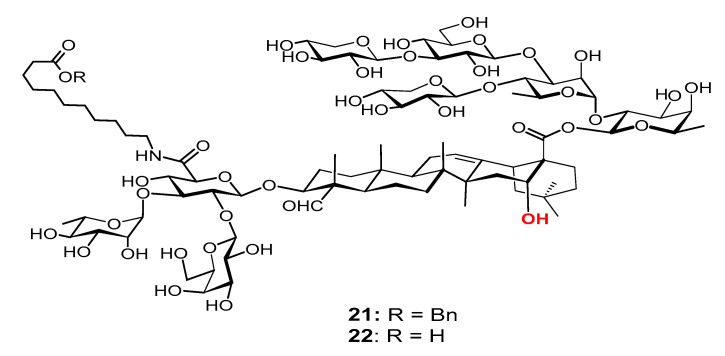

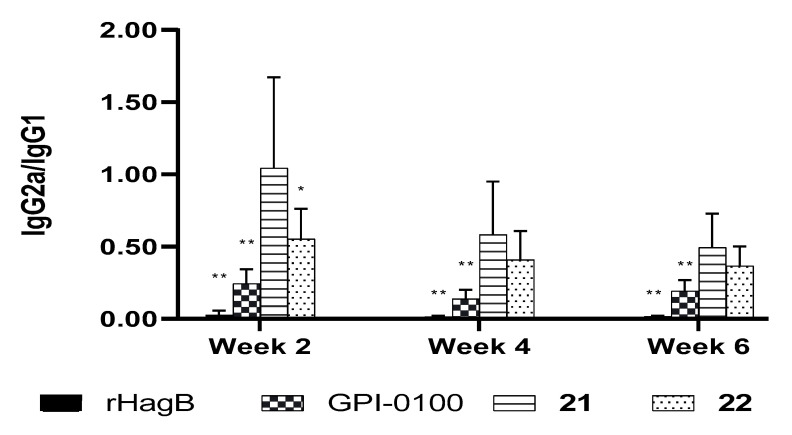
Semi-synthetic MS derivative VSA-2. Mice were immunized by the s.c. route with rHagB alone or with GPI-0100 or a saponin adjuvant on days 0, 14, and 28. Serum samples were collected prior to each immunization and at 6 weeks after the initial immunization. IgG2a/IgG1. Values are expressed as mean ± SD. Statistical significance compared with rHagB + VSA-2 (**21**). Statistical significance was evaluated by *t* tests (with unpaired, nonparametric, and Mann−Whitney test). * *p* < 0.05; ** *p* < 0.01.

Among the studied *MS* derivatives, VSA-2 (**21**, Figure 8), a derivative of *MS* II through incorporating11-aminoundecanoic acid benzyl ester as side chain, showed a consistently higher IgG2a/IgG1 ratio than that of GPI-0100 [119] and QS-21 (unpublished results). Interestingly, with this same side chain, the *MS* I and *MS* II derivatives demonstrated dramatically different adjuvant activities; the *MS* I derivative showed much lower IgG2a titers than both **19** (VSA-1) and **21** (VSA-2) with similar overall IgG titers. Again, these results suggest that a saponin’s adjuvant property is probably determined by its specific molecular structure (including the side chain structure), not by its overall amphipathicity, HLB, or the presence/absence of a particular functional group.

**Figure 9:**
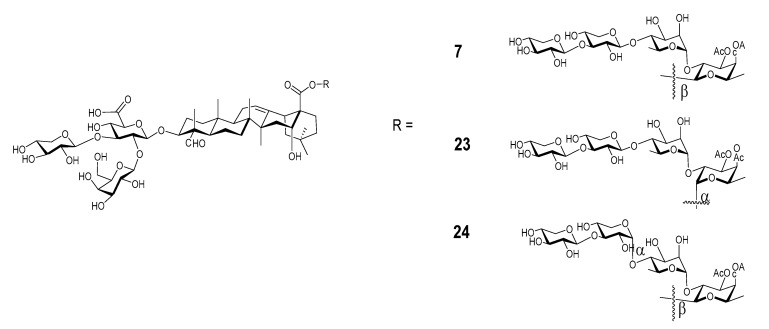
Synthetic *QS* isomeric analogs having different stereocenters.

Wang et al. recently synthesized and evaluated another series of *QS* derivatives containing saponin **7**, **23**, and **24** (Figure 9). These three synthetic saponins are stereoisomers, with **7** mimic natural QS-7 and QS-21. Compared with the structure of QS-21, synthetic saponin **7** does not have the characteristic acyl side chain; instead, it has two acetyl groups at 3-O and 4-O of the C28 fucosyl unit. The isomeric saponin **23** differs from **7** only in the stereochemisty of the glycosidic ester bond between the tetrasaccharide and the C28 carboxyl group of quillaic acid core, with **7** having a beta bond as in natural QS-7 and QS-21, but **23** having an unnatural alpha bond. Another stereisomer, **24**, only differs from **7** in the stereochemistry of an internal glycosidic bond as indicated in Figure 9. Interestingly, saponin **7** showed dramatically different adjuvant activity from the other two stereoisomers. Saponins **23** and **24** completely lost their capacity in enhancing antigen-specific IgGs, while saponin **7** remained highly active as an immunostimulant [112]. The observed loss of adjuvanticity due to the unnatural glycosidic ester bond between the tetrasaccharide domain and quillaic acid core is consistent with recent results of Ragupathi and Tan et al. in the study of their synthetic *QS* analogs [121]. As shown in the case of **24**, change of stereochemistry within the C28 oligosaccharide domain would also cost the saponin’s adjuvant activity. Moreover, changing the size of oligosaccharide [112] or replacing the original sugar units with structurally similar alternatives [122] in the synthetic analogs resulted in their significant loss of adjuvant activity. Thus, the role of the oligosaccharide is clearly not for maintaining a desirable HLB, and it is more likely for specific molecular recognition that is crucial to adjuvant activity.

Recent SAR studies not only provide mechanistic clues, suggesting saponins‘ adjuvant activity originated more likely from their interaction with specific molecular receptors than from their amphipathicity, but also lead to some practical alternatives to QS-21, e.g., the more accessible VSA-1 and VSA-2 adjuvants. The available toxicity data show that VSA-1′s toxicity is similar to that of GPI-0100, much less toxic than the widely used natural saponins [123]. While injected in 0.1 mL of PBS (BALB/c, female, 10 weeks of age) via the subcutaneous route, all of the mice in the groups treated with VSA-1 (2000 μg) survived, and the groups with VSA-1 (5000 μg) or *Quil* A (100 μg) reached endpoints within five days post injection that required euthanasia. *Quil* A, a purified mixture of *QS* saponins containing QS-21, has a similar adjuvant activity to QS-21 in potentiating a mixed Th1/Th2 immunity and CTL production. The surviving mice all had healthy looking fur and appeared to be behaving normally. None of the surviving mice seemed lethargic in any way by day 7, and no lesion formation was observed on any of the mice. These data show that the acute toxicity of VSA-1 was much lower than that of *Quil* A (which has a toxicity profile similar to that of QS-21, and the recommended dose of QS-21 for mice is no more than 20 μg). Recent studies showed that VSA-2 also had low toxicity (unpublished). In ongoing evaluations of VSA adjuvants in different experimental vaccines, VSA (at dose of 50 μg/mice) showed better activity than the established clinical and experimental adjuvants such as QS-21, CpG+MPL, alum, and Freund’s adjuvant (unpublished).

## 5. Conclusions

Saponins, including QS-21, could play a multifaceted role in orchestrating an effective and sophisticated antigen-specific immune response. The amphipathicity could probably contribute to, as suggested, facilitating cholesterol-mediated immune cell uptakes and other non-receptor-mediated process. However, SAR studies indicate the unique adjuvant activity of QS-21 should be attributed more to its interaction with specific molecular targets, given significant stimulating activity profile change with subtle change of the molecular structure even when the HLB of the molecules remain largely unchanged. Given the sensitivity of the structure-dependent adjuvant activity, the mechanistic insights obtained with a saponin probe equipped with a large imaging group might not be extrapolated to QS-21 unless the probe’s adjuvant activity/toxicity profiles closely resemble that of QS-21.

## Data Availability

The data presented in this study are available on request from the corresponding author.
